# Editorial: Nutritional and physical activity strategies to boost immunity, antioxidant status and health, Volume III

**DOI:** 10.3389/fphys.2023.1199066

**Published:** 2023-04-20

**Authors:** Mallikarjuna Korivi, Arifullah Mohammed, Weibing Ye, Veeranjaneya Reddy Lebaka

**Affiliations:** ^1^ Institute of Human Movement and Sports Engineering, College of Physical Education and Health Sciences, Zhejiang Normal University, Jinhua, China; ^2^ Department of Agriculture Science, Faculty of Agro-Based Industry, Universiti Malaysia Kelantan, Jeli, Kelantan, Malaysia; ^3^ Department of Microbiology, Yogi Vemana University, Kadapa, India

**Keywords:** exercise, nutritional supplement, skeletal muscle function, aging, overall health

This Research Topic comprised of research articles, reviews, and meta-analyses, emphasizes the beneficial effects of exercise or food-based interventions alone or in combination on skeletal muscle function, antioxidant status and wellbeing in both humans and animal models. Intake of various plant-based supplements from fruit, root or leaves has been shown to promote overall health against various diseases. For instance, supplementation of *Spondias mombin* leaves extract for 28 days exhibits antidiabetic property through decreased blood glucose and restored insulin levels in diabetic rats. This hypoglycemic effect was further accompanied by alleviation of dyslipidemia and improvement of liver function. The phytochemical in *Spondias mombin* leaves, namely flavonoids, anthraquinone, tannins, saponins, steroids, phenols and alkaloids may be contributed to these beneficial effects Gobinath et al. Another laboratory study stated that treatment of *Solanum torvum* fruit extracts to diabetic rats considerably reversed the hyperglycemia and dyslipidemia. Besides, this fruit extract decreased the elevated liver transaminases, and increased the number and size of pancreatic β-cells against streptozotocin-induced destruction Satyanarayana et al. These reports suggest that supplementation of plant-based extracts can effectively attenuate the diabetes-induced complications, and thereby protect the tissues.

Sarcopenia, loss of muscle mass and strength due to natural aging is an inevitable phenomenon leading to decline the quality of life among older people. Therefore, it is necessary to find out the alternative strategies to maintain the physical fitness and improve overall health of aged population. One of the best ways to delay the sarcopenia or improve fitness is regular exercise with or without proper dietary intake. In an aging study on rats, Su et al., demonstrated that age-induced progressive loss of skeletal muscle mass index and muscle fiber cross section area (CSA) was reversed by 32-week high-intensity interval training (HIIT) and resistance training (RT). The reactive oxygen species (ROS), which play a critical role in propagation of age-induced apoptosis, were found to be lower in the soleus of aging rats after HIIT. Besides, both HIIT and RT modalities attenuated the age-associated pro-apoptotic signals Su et al. Another study reported that RT combined with isolated soy protein supplementation had greater beneficial effects on physical performance, muscle strength and muscle glycogen reserves in aging mice. The increased type II muscle fibers and CSA further evidenced that combination of soy protein plus RT had greater beneficial effect than that of soy protein or RT intervention alone. These results indicate that age-induced loss of muscle mass could be maintained and/or delayed through exercise plus soy protein supplementation Lee et al. Although exercise is effective in improving skeletal muscle mass and physical fitness, the repeated high-intensity exercise-induced oxidative stress, inflammation or muscle damage cannot be ruled-out when prescribing exercise for older adults. In view of this, quercetin, a phytochemical flavonoid has been tested for its beneficial effects against high-intensity exercise in healthy adults. Tsaoet al. reported that short-term quercetin supplementation (7 days) effectively improved the time-to-exhaustion of high-intensity cycling exercise in healthy adults. This beneficial effect of quercetin may be due to the increased glucose uptake and/or attenuation of high-intensity exercise-induced oxidative stress and pro-inflammatory response Tsaoet al. These findings emphasizes that exercise training combined with nutritional supplements are effective in improving the skeletal muscle function, endurance performance and antioxidant capacity.

A study on young male athletes examined the muscle damaging effects of acute RT under normoxia and hypoxia-hyperoxia conditions. This study reported higher muscle soreness, increased creatine kinase and myoglobin levels after acute RT under normoxia. However, athletes exposed to hypoxia-hyperoxia reported no such adverse effects following acute RT. These findings imply that hypoxia-hyeroxia alternative exposure prior to intense exercise can alleviate the muscle damage and muscle soreness in athletes Chen et al. On the other hand, blood flow restriction (BFR), which increases the mechanical pressure to working muscle during exercise, can cause local hypoxia/ischemia. In this context, a systematic review and meta-analysis summarized that exercise with BFR can increase the mRNA expressions of angiogenic factors, including vascular endothelial growth factor (VEGF) and hypoxia-inducible factor-1α (HIF-1α) in healthy adults. To be specific, RT combined with BFR appears to be greater in promoting the skeletal muscle angiogenic factors (VEGF and HIF-1α) than aerobic exercise with BFR Li et al. Other than exercise-induced beneficial effects on skeletal muscle in healthy adults, it is important to know the influence of exercise on improving the balance capability in children/adolescents with hearing impairment. To address this, a systematic review included eligible studies which examined the intervention effect on balance ability of children and adolescent with hearing impairment. This systematic review concluded that exercise intervention is effective in improving the balance among children/adolescents with hearing impairments. Specifically, intervention with a duration of 8–16-week is more efficient than a duration of < 8-week in improving the balance ability Zhou et al. Depression is a worldwide health issue for people that commonly coexist with other debilitating chronic illnesses. The correlation between depression and low-levels of vitamin D is still debatable. A meta-analysis synthesized evidence from randomized controlled trials, and stated that vitamin D supplementation is not only beneficial to decline the incidence of depression, but also effective to treat depression. It is further emphasized that supplementation of vitamin D with a daily dose of >2,800 IU, and intervention duration of 8-week or more were effective in prevention and treatment of depression Xie et al. Our Research Topic highlights that exercise training with or without combination of nutritional supplements is capable of improving the skeletal muscle function, antioxidant homeostasis and maintain overall wellbeing.

